# Comparative Genomics Analysis of *Mycobacterium ulcerans* for the Identification of Putative Essential Genes and Therapeutic Candidates

**DOI:** 10.1371/journal.pone.0043080

**Published:** 2012-08-13

**Authors:** Azeem Mehmood Butt, Izza Nasrullah, Shifa Tahir, Yigang Tong

**Affiliations:** 1 National Centre of Excellence in Molecular Biology (CEMB), University of the Punjab, Lahore, Pakistan; 2 Department of Biochemistry, Faculty of Biological Sciences, Quaid-i-Azam University, Islamabad, Pakistan; 3 National Center for Bioinformatics, Faculty of Biological Sciences, Quaid-i-Azam University, Islamabad, Pakistan; 4 State Key Laboratory of Pathogen and Biosecurity, Beijing Institute of Microbiology and Epidemiology, Beijing, People's Republic of China; New England Biolabs, United States of America

## Abstract

*Mycobacterium ulcerans*, the causative agent of Buruli ulcer, is the third most common mycobacterial disease after tuberculosis and leprosy. The present treatment options are limited and emergence of treatment resistant isolates represents a serious concern and a need for better therapeutics. Conventional drug discovery methods are time consuming and labor-intensive. Unfortunately, the slow growing nature of *M. ulcerans* in experimental conditions is also a barrier for drug discovery and development. In contrast, recent advancements in complete genome sequencing, in combination with cheminformatics and computational biology, represent an attractive alternative approach for the identification of therapeutic candidates worthy of experimental research. A computational, comparative genomics workflow was defined for the identification of novel therapeutic candidates against *M. ulcerans*, with the aim that a selected target should be essential to the pathogen, and have no homology in the human host. Initially, a total of 424 genes were predicted as essential from the *M. ulcerans* genome, via homology searching of essential genome content from 20 different bacteria. Metabolic pathway analysis showed that the most essential genes are associated with carbohydrate and amino acid metabolism. Among these, 236 proteins were identified as non-host and essential, and could serve as potential drug and vaccine candidates. Several drug target prioritization parameters including druggability were also calculated. Enzymes from several pathways are discussed as potential drug targets, including those from cell wall synthesis, thiamine biosynthesis, protein biosynthesis, and histidine biosynthesis. It is expected that our data will facilitate selection of *M. ulcerans* proteins for successful entry into drug design pipelines.

## Introduction

*Mycobacterium ulcerans* is the etiologic agent of Buruli ulcer (BU), a quickly emerging yet neglected infectious tropical disease characterized mainly by chronic necrotizing skin ulcers. It is presently the third most common mycobacterial human disease, after tuberculosis and leprosy [1]. BU is found mostly in West Africa, but during the past decade its rate of incidence has increased dramatically in the tropical and subtropical regions of Asia, the Western Pacific, and Latin America [2]. It has been estimated that *M. ulcerans* diverged from a fish pathogen, *Mycobacterium marinum*, which is also able to cause granulomatous skin infections in humans. The divergence event has been estimated to have occurred between 470,000 and 1,200,000 years ago, as evidenced by the acquisition of the 174 kb virulence plasmid pMUM001 by *M. ulcerans* [3], [4]. Acquisition of this plasmid is believed to be responsible for the severe nature of *M. ulcerans*, as this plasmid harbors a cluster of genes necessary for synthesis of the polyketide toxin mycolactone. This toxin appears largely responsible for the massive tissue destruction seen in BU patients [5]. Unfortunately, despite its increasing prevalence around the globe, the epidemiology, mode of transmission, and molecular mechanisms of *M. ulcerans* and the associated disease remain poorly understood. The current World Health Organization approved standard treatment for *M. ulcerans* is an eight week course of rifampicin plus streptomycin (R + S) chemotherapy. However, antibiotic treatment is only effective in the early stages of infection and surgical excision is the only option left for most patients in advanced stages [6]. The emergence of antibiotic resistant *M. uclerans* strains has also been reported [7]. This strongly indicates there is continuous need to search for additional drug targets in the bacterial genome that would offer better protection and less long-term resistance. In addition, a controlled combination of multiple drugs is more desirable for effective treatment outcomes.

Although the experimental verification of drug targets cannot be replaced, obstacles include expense, time, and the slow growth rates and cultural difficulties of many bacterial species. Unfortunately *M. ulcerans* is a very slow-growing bacterium, requiring up to three months of incubation at 32°C to form countable colonies on solid media [8]. As BU is still mostly a disease of rural areas, is a neglected tropical disease, and is difficult to culture, *M. ulcerans* has not received much attention from the pharmaceutical industry; with the result that treatment options are limited. To speed up the discovery process and increase treatment options, there is a need to find alternative ways to identify drug and vaccine candidates. The search for drug targets using computational methods and integrated “omics” data, such as genomics, proteomics, and metabolomics, has received much attention in the past few years and these research areas are expanding. Comparative genomics, differential genomics, and subtractive genomics have emerged as widely used approaches for the identification of potential therapeutic candidates in numerous pathogenic bacteria and fungi [9]–[13]. In principle, these approaches rely on searching for those genes/proteins that are absent in the host but present in the pathogen. Furthermore, these non-host genes must be essential for the survival of the pathogen, and be critical components in vital physicochemical and metabolic pathways. A designed drug or lead compound should thus target only the pathogen’s system, without affecting the physiology or biology of the host. It is expected that use of full genome sequences to find genetic content essential for bacterial survival and pathogenicity, along with modern bioinformatics algorithms and approaches, can greatly reduce the time required searching for novel therapeutic targets. The most common mechanism of antibiotics is to inhibit targeted bacterial enzymes. Theoretically all enzymes specific to bacterial systems can be considered as potential drug targets [14].

In genomics-based drug discovery it is important to have information about a minimal genome set or essential genes, because the essential gene products of pathogenic bacteria are attractive drug targets for antibiotic development, and also highlight fundamental life-support functions. However, similarly to methods of conventional drug discovery, experimental identification of essential genes via methods such as single gene knockouts, RNA interference, and conditional knockouts is labor-intensive, expensive, and time-consuming. Although methods of genome-wide essential gene identification have improved significantly over the past few years, they are still unable to maintain pace with the amount of data appearing from full genome sequencing projects. Experimentally determined essential genome content has been reported to date from 20 bacteria; in contrast, the latest update of the National Center for Biotechnology Information (NCBI) genome database contains more than 1000 complete bacterial genomes. To compensate for this huge gap, many studies around the globe have focused on developing alternative computational methods for identification of essential genes in bacteria of interest. Analyses of available genetic data have revealed that there are several unique characteristics that distinguish essential genes from non-essential. These include a higher rate of evolutionary conservation, strand-bias, different patterns of protein interaction networks, high expressivity, codon usage, GC content, length of proteins, and subcellular localization [15]–[18]. Modern computational biology has successfully incorporated several of these unique genomic features, and devised algorithms and data-mining methods for computational identification of essential genes. While every method has advantages and limitations, among the sequence-derived methods, analysis of gene conservation among closely related and even in distantly related species via homology searching, has been the best predictor and most widely used method for essential genes identification. This has been used for more than 30 bacteria, including *Mycobacterium leprae* [19], *Burkholderia pseudomallei* [20], *Staphylococcus aureus* [21], *Pseudomonas aeruginosa* [10], *Leptospira interrogans* [22], and *Wolbachia* [23].

The first complete 5.8 Mb genome of *M. ulcerans* strain Agy99, isolated from Ghana, was sequenced in 2007 [4]. It is now publically available; representing an excellent opportunity to computationally predict essential genes and associated metabolic pathways; thus accelerating drug discovery steps. We studied the *M. ulcerans* genome with two objectives; identification of putative essential genes and comparative genomics analysis of metabolic pathways of the pathogen and host for the identification of therapeutic candidates. It is expected that the identified targets will expand our understanding of the molecular mechanisms of *M. ulcerans* pathogenesis, and facilitate the production of novel therapeutic agents.

## Materials and Methods

### Prediction of Essential Genes

The genomic RefSeq protein sequences of *M. ulcerans* Agy99 strain (RefSeq: NC_008611.1) were retrieved from the NCBI genome database (ftp://ftp.ncbi.nih.gov/genomes/Bacteria). The latest update (version 6.8; November 4, 2011) of the Database of Essential Genes (DEG) [24], compiles literature and sequences of experimentally verified essential genes and proteins from 20 Gram-positive and Gram-negative bacteria (Table S1). This was downloaded from the DEG website (http://tubic.tju.edu.cn/deg/deg.rar). The standalone release of NCBI BLASTP+ version 2.2.26 was obtained from the NCBI ftp site (ftp://ftp.ncbi.nlm.nih.gov/blast/executables/blast/LATEST/). BLASTP was installed on a local machine and a search was performed to align the *M. ulcerans* protein sequences against the essential protein sequences obtained from the DEG. Criteria for short listing of essential proteins/genes were as follows; expect value (E-value) cut-off of 10^−10^, a minimum bit score of 100, and percentage of identity ≥35% between query and hits.

### Prediction of the Therapeutic Targets

**Figure 1:**
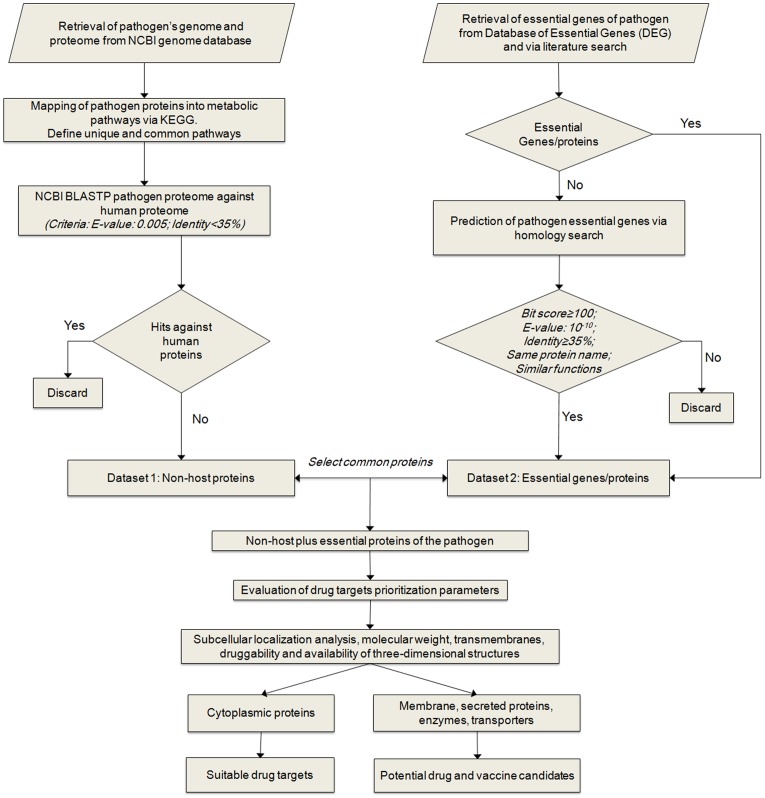
Comparative genomics workflow. Overview of steps involved in computational comparative genomics-based target identification and essential genes in *M. ulcerans*. Identified targets can be used to develop drugs or vaccines, depending on their non-host plus essential nature, associated metabolic pathways, and drug targets prioritization parameters.

The workflow of comparative genomics was defined for the prediction of therapeutic targets against *M. ulcerans*. The workflow (Figure 1) comprises several steps in the selection of attractive drug targets, as discussed below.

#### Analysis of host and pathogen metabolic pathways

Genome-wide metabolic pathway analysis was performed via the Kyoto Encyclopedia of Genes and Genomes (KEGG) database (http://www.genome.jp/kegg/) [25], [26]. Metabolic pathways and assigned identification numbers of the pathogen *M. ulcerans* and the human host were extracted from the KEGG database. A manual comparison was then made, and pathways that did not appear in the host but were present in the pathogen, according to the KEGG database annotations, were selected as unique to *M. ulcerans*, while the remaining pathways were listed as common. *M. ulcerans* proteins from common and unique pathways were then identified, and the respective amino acid sequences were obtained from the NCBI genome database.

#### Identification of non-host and non-host plus essential proteins

Two-step comparisons were performed between host and pathogen proteomes for the identification of non-host proteins from *M. ulcerans*. At first, only proteins from pathogen-specific pathways were subjected to NCBI BLASTP analysis [27]. Second, proteins from common pathways were also compared by BLASTP analysis. In each scenario, searching was restricted to proteins from *H. sapiens* (taxid: 9606), through an option available under the NCBI BLASTP parameters. Proteins without hits below the E-value inclusion threshold of 0.005 and <35% identity, were chosen as non-host bacterial proteins. Once all the non-host proteins were identified, they were further filtered on the basis of essentiality by comparison with protein sequences of the *M. ulcerans* essential genes for the creation of a non-host essential proteins dataset.

#### Prioritization parameters for therapeutic targets

Several molecular and structural criteria that have been proposed to aid in prioritizing suitable therapeutic targets in pathogenic microorganisms [28] were also evaluated for each of the predicted therapeutic targets in *M. ulcerans*. This involved, calculation of molecular weight (MW) using computational tools and drug target-associated literature available in the Swiss-Prot database [29]. Prediction of biological significance and subcellular localization of therapeutic targets was carried out using MycobacSVM (http://211.83.105.213/server/MycobacSVM/MycobacSVM.htm) [30]. MycobacSVM is a support vector machine based method that uses feature selection techniques to specifically predict subcellular location of mycobacterial proteins at four levels: cytoplasmic, integral membrane, secretory, and membrane attached proteins by a lipid anchor. Transmembrane predictions were made using TMHMM v2.0 (http://www.cbs.dtu.dk/services/TMHMM/) [31] and the TOCONS consensus membrane proteins topology prediction server, which combines predictions from four different algorithms (http://topcons.cbr.su.se/) [32]. Experimentally and computationally solved 3D structures were detected by searching the Protein Data Bank (PDB) (http://www.rcsb.org/pdb) [33] and ModBase (http://salilab.org/modbase) [34] databases, respectively.

#### Druggability

Druggability is another important therapeutic prioritization criterion; defined as the likelihood of being able to modulate the activity of the target protein with a small-molecule drug [35], [36]. The druggability potential of each identified drug target was calculated by mining the DrugBank contents (http://www.drugbank.ca/). The DrugBank database is a unique bioinformatics and cheminformatics resource that combines detailed drug (i.e. chemical, pharmacological and pharmaceutical) data with comprehensive drug target (i.e. sequence, structure, and pathway) information. The database contains 6,711 drug entries, including 1,441 FDA-approved small molecule drugs, 134 FDA-approved biotech (protein/peptide) drugs, 84 nutraceuticals, and 5,084 experimental drugs. In addition, 4,231 non-redundant protein (i.e. drug target/enzyme/transporter/carrier) sequences are linked to these drug entries [37]. A BLASTP with default parameters was performed to align the potential drug targets from *M. ulcerans* against the list of protein targets of compounds found within DrugBank. The selection criteria for filtering BLAST results were as described previously for identification of drug targets in bacterial genomes [23], that is, alignments with e-values less significant than 1×10^−25^ were removed.

## Results and Discussion

### Metabolic Pathways Analysis and Identification of Non-host Proteins

Here we report the first computational comparative genomics analysis of *M. ulcerans* aimed at the identification of potential therapeutic candidates. A systematic workflow was defined that involved several bioinformatics tools, databases, and drug target prioritization parameters (Figure 1). Initial information about the metabolic pathways of *M. ulcerans* and its human host was derived from the KEGG database. The KEGG presently contains information about 103 metabolic pathways in *M. ulcerans* Agy99 and 110 in *H. sapiens*. Names and total numbers of proteins present in each pathway were calculated, and a manual two-list comparative analysis was performed for the identification of pathways specific to *M. ulcerans*, and pathways common to *M. ulcerans* and *H. sapiens*. Twenty-nine different metabolic pathways were identified as unique to *M. ulcerans*, and 74 pathways were shared (Table 1).

**Table 1:** Unique metabolic pathways of *M. ulcerans* and pathways common to *M. ulcerans* and humans based on KEGG annotations.

No	Unique Pathways	Pathways ID	Total Proteins
01	C5-Branched dibasic acid metabolism	00660	04
02	Methane metabolism	00680	20
03	D-Alanine metabolism	00473	02
04	Peptidoglycan biosynthesis	00550	14
05	Limonene and pinene degradation	00903	10
06	Geraniol degradation	00281	07
07	Polyketide sugar unit biosynthesis	00523	04
08	Biosynthesis of siderophore group nonribosomal peptides	01053	09
09	Penicillin and cephalosporin biosynthesis	00311	02
10	Streptomycin biosynthesis	00521	08
11	Novobiocin biosynthesis	00401	02
12	Benzoate degradation	00362	11
13	Aminobenzoate degradation	00627	08
14	Fluorobenzoate degradation	00364	01
15	Chloroalkane and chloroalkene degradation	00625	04
16	Chlorocyclohexane and chlorobenzene degradation	00361	05
17	Toluene degradation	00623	03
18	Xylene degradation	00622	02
19	Nitrotoluene degradation	00633	02
20	Ethylbenzene degradation	00642	02
21	Styrene degradation	00643	01
22	Atrazine degradation	00791	03
23	Caprolactam degradation	00930	04
24	Bisphenol degradation	00363	05
25	Dioxin degradation	00621	03
26	Naphthalene degradation	00626	07
27	Polycyclic aromatic hydrocarbon degradation	00624	03
28	Bacterial secretion system	03070	12
29	Two-component system	02020	22
**No**	**Common Pathways**	**Pathways ID**	**Total Proteins**
01	Glycolysis/Gluconeogenesis	00010	21
02	Citrate cycle (TCA cycle)	00020	14
03	Pentose phosphate pathway	00030	16
04	Pentose and glucuronate interconversions	00040	04
05	Fructose and mannose metabolism	00051	09
06	Galactose metabolism	00052	07
07	Ascorbate and aldarate metabolism	00053	02
08	Starch and sucrose metabolism	00500	14
09	Amino sugar and nucleotide sugar metabolism	00520	20
10	Pyruvate metabolism	00620	21
11	Glyoxylate and dicarboxylate metabolism	00630	11
12	Propanoate metabolism	00640	14
13	Butanoate metabolism	00650	15
14	Inositol phosphate metabolism	00562	05
15	Oxidative phosphorylation	00190	41
16	Nitrogen metabolism	00910	12
17	Sulfur metabolism	00920	09
18	Fatty acid biosynthesis	00061	07
19	Fatty acid metabolism	00071	13
20	Synthesis and degradation of ketone bodies	00072	03
21	Steroid biosynthesis	00100	03
22	Glycerolipid metabolism	00561	06
23	Glycerophospholipid metabolism	00564	12
24	Ether lipid metabolism	00565	02
25	Alpha-Linolenic acid metabolism	00592	02
26	Biosynthesis of unsaturated fatty acids	01040	01
27	Purine metabolism	00230	43
28	Pyrimidine metabolism	00240	25
29	Alanine, aspartate and glutamate metabolism	00250	20
30	Glycine, serine and threonine metabolism	00260	19
31	Cysteine and methionine metabolism	00270	19
32	Valine, leucine and isoleucine degradation	00280	15
33	Valine, leucine and isoleucine biosynthesis	00290	11
34	Lysine biosynthesis	00300	15
35	Lysine degradation	00310	07
36	Arginine and proline metabolism	00320	23
37	Histidine metabolism	00340	12
38	Tyrosine metabolism	00350	08
39	Phenylalanine metabolism	00360	07
40	Tryptophan metabolism	00380	09
41	Phenylalanine, tyrosine and tryptophan biosynthesis	00400	16
42	Beta-Alanine metabolism	00410	09
43	Taurine and hypotaurine metabolism	00430	04
44	Selenocompound metabolism	00450	07
45	Cyanoamino acid metabolism	00460	04
46	D-Glutamine and D-glutamate metabolism	00471	04
47	D-Arginine and D-ornithine metabolism	00472	01
48	Glutathione metabolism	00480	06
49	Thiamine metabolism	00730	08
50	Riboflavin metabolism	00740	07
51	Vitamin B6 metabolism	00750	05
52	Nicotinate and nicotinamide metabolism	00760	11
53	Pantothenate and CoA biosynthesis	00770	13
54	Biotin metabolism	00780	06
55	Lipoic acid metabolism	00785	02
56	Folate biosynthesis	00790	11
57	One carbon pool by folate	00670	11
58	Porphyrin and chlorophyll metabolism	00860	29
59	Ubiquinone and other terpenoid-quinone biosynthesis	00130	07
60	Terpenoid backbone biosynthesis	00900	17
61	RNA polymerase	03020	04
62	Ribosome	03010	36
63	Aminoacyl-tRNA biosynthesis	00970	19
64	Protein export	03060	14
65	Sulfur relay system	04122	08
66	Proteasome	03050	03
67	RNA degradation	03018	08
68	DNA replication	03030	12
69	Base excision repair	03410	12
70	Nucleotide excision repair	03420	08
71	Mismatch repair	03430	05
72	Homologous recombination	03440	15
73	Non-homologous end-joining	03450	02
74	ABC transporters	02010	34

The next step was to find non-host proteins from the *M. ulcerans* genome. The term “non-host” refers here to those bacterial proteins that show no homology with human proteins. It has been suggested that such proteins can serve as better drug targets, in terms of avoiding the likely side effects and cross-reactivity caused by antibiotics. Selection of non-host proteins from bacterial genomes remains therefore a critical step in computational drug discovery. For the identification of such proteins in the *M. ulcerans* genome, amino acid sequences of protein-coding genes from common and unique metabolic pathways were obtained from the KEGG and NCBI databases, and compared with the human proteome using NCBI BLASTP. A total of 411 proteins from the *M. ulcerans* genome showed “no hits” against the human proteome and were classified as non-host proteins. Among these, 87 were associated with unique metabolic pathways and 324 with common pathways (Table S2). Initially, this information regarding non-host proteins and their metabolic pathways can be used and has been previously used for the prediction of drug targets. However, to minimize the time required for drug testing and development, the inclusion of gene essentiality information and drug prioritization parameters offers great advantage in the careful selection of candidates for drug discovery pipelines [13].

### Essential Genes of *M. ulcerans*

Here, we report the computational identification of putative essential genes of *M. ulcerans* via the homology search method. Essential genes predictions from the individual features of essential genes and from algorithms that combine several of these features, have shown significant sensitivity and accuracy when applied to experimentally verified essential genes as training and verification datasets [16], [18]. A detailed evaluation of these computational algorithms, in parallel with experimental verification, has yet to be done. Also, in many instances, prediction classifiers trained on one species dataset do not produce the same prediction accuracy when applied to other bacterial genomes. The predictive potential of homology searching, based on gene conservation and common essential genes, was made apparent in a recent study of essential gene identification in *Yersinia pseudotuberculosis* [38]. Here, 7 of 8 computationally predicted essential genes via DEG-based homology search, were also experimentally validated as essential for *Y. pseudotuberculosis*. Furthermore, these essential genes were identified as essential and conserved across more than nine bacterial genomes that were present in the DEG at the time of study [38].

In general, essential gene prediction via homology searching is based on the notion that a query gene is likely to be essential if its homolog is present in another bacterium as an experimentally validated essential gene. It can be expected, therefore, that as more information regarding essential bacterial genes becomes available from experimental studies, prediction results will increase in accuracy, including in genomes for which experimental approaches have not yet been conducted or are difficult to perform. Therefore, by taking advantage of essential genes information from 20 different bacteria (Table S1), we report 424 protein-coding genes from the *M. ulcerans* genome as essential via DEG based homology search and following selection criteria of E-value cut-off of 10^−10^, a minimum bit score of 100, and percentage of identity ≥35% between query and hits. These predicted essential genes are listed in Table S3, along with the names and associated DEG IDs of bacteria that were the first best hits against query sequences from the *M. ulcerans* genome. As per NCBI genome annotation information available for the *M. ulcerans* Agy99 strain, the total gene products or proteins are 4,241. Following our genome-wide analysis, a total of 424 genes/proteins were predicted as essential out of the total 4,241 and therefore the total *M. ulcerans* proteome predicted as essential is 10% (Table S3).

**Figure 2:**
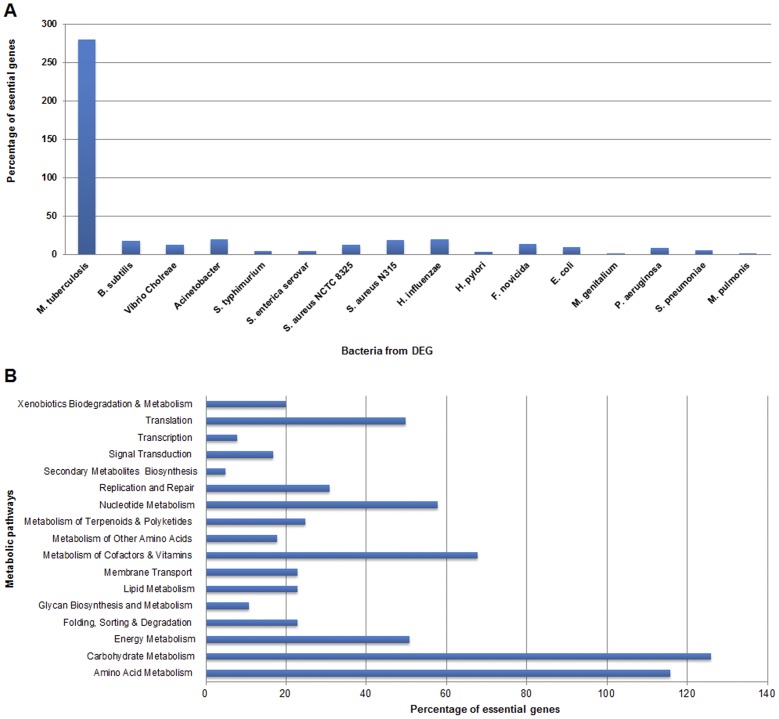
Homology search and metabolic pathways analysis of *M. ulcerans* essential genes. (**A**) Essential genes of *M. ulcerans* having homology to essential genes from other bacteria. (**B**) Percentage distribution of *M. ulcerans* essential genes into associated metabolic pathways.

As stated earlier, the recent update of the DEG contains experimentally verified essential genes from 20 different bacteria. We further evaluated the number of essential genes that *M. ulcerans* shares with other bacteria. The most essential gene matches came from *M. tuberculosis* (279 out of 424) and the fewest from *Mycoplasma genitalium* (1 out of 424) and *M. pulmonis* (1 out of 424) (Figure 2A and Table S3). As *M. ulcerans* and *M. tuberculosis* are both *Mycobacteria*, many of conserved genes were expected. *M. tuberculosis* is the only mycobacterium to date for which gene essentiality studies have been conducted in comparison with 34 other *Mycobacteria* whose full genomes have been sequenced and are publically available in the NCBI genome database. In our opinion, better results can be obtained from the availability of essential genes information from other mycobacterial species; particularly from *M. marium*, the ancestor of *M. ulcerans*. Although not the focus of our study, it would be interesting to perform a DEG-based essential gene prediction for *M. marium*, and then compare common essential genes between *M. marium* and *M. ulcerans*. Currently, 614 genes of *M. tuberculosis* are known to be essential. However, only 279 showed considerable identity with *M. ulcerans* genes as per our selection criteria. It can be hypothesized therefore, that the common 279 essential genes are the core-essential genes i.e. those common among all mycobacterial species. However, this requires further investigation.

### Association of Essential Genes with Metabolic Pathways

Once the essential genes of *M. ulcerans* were predicted, we examined the distribution and association of essential genes in metabolic pathways. In accordance with the KEGG database annotations, the essential genes mapped to 17 metabolic pathway categories. The categories of replication and repair, translation, energy metabolism, nucleotide metabolism, metabolism of cofactors and vitamins, amino acid metabolism, and carbohydrate metabolism had the highest numbers of essential genes (Table S3). We next investigated the distribution of essential genes into the biochemical pathways within each category. The essential genes were clustered within many pathways. The most notable were: (i) glycolysis, (ii) the pentose phosphate pathway, (iii) the citrate cycle, (iv) terpenoid backbone biosynthesis, (v) glycerophospholipid metabolism, (vi) glycerolipid metabolism, (vii) fatty acid biosynthesis, (viii) nucleotide biosynthesis, (ix) folate biosynthesis, (x) thiamine metabolism, (xi) oxidative phosphorylation, (xii) DNA replication, (xiii) transcription, (xiv) protein biosynthesis, and (xv) ABC transporters (Figure 2B and Table S3). Finally, the distribution of essential genes into unique and common metabolic pathways was inferred, and out of 424 essential genes, 73 were from unique and 351 were from common pathways (Table S3).

### Evaluation of Essential Genes Based on Prediction Features

Although homolog searching was our method of choice for prediction of essential genes, we were interested in evaluating how other features of essential genes identification corresponded with our results. For this purpose, we selected the following four features that can be analyzed from sequence data: (i) strand-bias; (ii) clusters of orthologous groups (COG) of proteins; (iii) the codon adaptation index (CAI); and (iv) patterns of enzyme classes distribution among essential genes.

#### Strand-bias among essential genes of *M. ulcerans*

**Figure 3:**
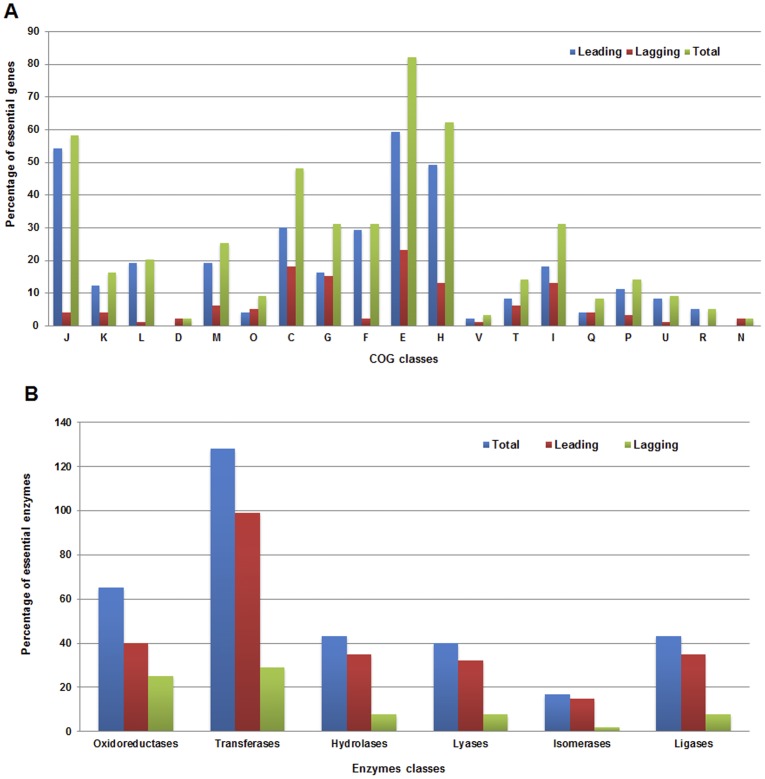
Evaluation of *M. ulcerans* essential genes via essential genes prediction features. (**A**) Percentage distribution of M. *ulcerans* essential genes on leading and lagging stands and among COG functional subcategories. (**B**) Percentage distribution of *M. ulcerans* essential genes on leading and lagging strands for six enzyme classes. Strand-bias towards different enzyme classes is also shown.

It is known that essential genes show strand bias and are preferentially located on leading strands rather than lagging strands. This phenomena was initially studied for *E. coli* and *B. subtilis* [15]; and it was postulated that essentiality, rather than expressivity, drives strand-bias. Recently, this phenomena of strand-bias among essential genes has been confirmed in two studies. In one, the authors calculated the biased distribution of essential genes on leading and lagging strands, by analyzing experimentally driven data of essential genes from ten bacterial species [39]. In the other, strand-bias was studied among essential genes from 16 different *Mycoplasma* species [40]. We analyzed strand-bias in the *M. ulcerans* genome at two levels, genome-wide and for the predicted set of essential genes. Initially, the replication origins and replication termini were predicted using Ori-Finder [41], based on which the bacterial genes located on the leading and lagging strands were determined. Among the 4,957 genes of *M. ulcerans*, 2,720 were identified on the leading strand and the 2,237 others on the lagging strand (data available on request). When the same was applied on the 424 essential genes of *M. ulcerans,* the distribution pattern and strand-bias became significant, as 319 essential genes were found on the leading strand and the remaining 105 were on the lagging strand (Figure 3A and Table S3); thus supporting leading strand-bias among essential genes of *M. ulcerans*. This and data from previous studies therefore favors that strand-bias can be effectively used as a selection parameter in essential gene prediction algorithms.

#### Essential genes of *M. ulcerans* are biased towards COG

Lin et al. has recently reported that essential genes are biased towards the following ten COG functional subcategories: J (translation, ribosomal structure and biogenesis), K (transcription), L (replication, recombination and repair), D (cell cycle control, cell division, chromosome partitioning), M (cell wall/membrane/envelope biogenesis), O (post-translational modification, protein turnover, chaperones), C (energy production and conversion), G (carbohydrate transport and metabolism), E (amino acid transport and metabolism), and F (nucleotide transport and metabolism) [39]. In parallel to this, Lin et al. also showed that essential genes from the above ten COG categories were preferentially located on leading strands rather than lagging strands. To test if this applies in *M. ulcerans*, COG functional classes were assigned to 424 essential genes via the NCBI COG database (http://www.ncbi.nlm.nih.gov/COG/). Where one essential gene was assigned to two or more COG functional categories, it was counted in each category, as previously described [39]. The essential genes of *M. ulcerans* were distributed in 19 COG categories, namely J,K,L,D,M,O,C,G,F,E,H,V,T,I,Q,P,U,R, and N (Figure 3A and Table S3). Most essential genes belonged to COG subcategory E, followed by H, and J. Subcategories D,V (defence mechanisms), and M (cell wall/membrane/envelope biogenesis) had the lowest numbers of essential genes (Figure 3A). None of the essential genes of *M. ulcerans* were mapped to subcategories B (chromatin structure and dynamics), Y (nuclear structure), Z (cytoskeleton), or W (extracellular structures). These findings are in agreement with the work of Lin et al., with a few exceptions, such as that the *M. ulcerans* essential genes from COG subcategories D, O, and G did not show significant differences between leading and lagging strands. In addition, essential genes from subcategories P (inorganic ion transport and metabolism), and U (intracellular trafficking, secretion, and vesicular transport) also showed bias towards the leading strand. It is well known that highly-expressed essential genes are preferentially situated on the leading strand in order to avoid head-on collisions between DNA and RNA polymerases [15]. This analysis can be applied to the functional subcategory J, because many genes from this subcategory code for ribosomal proteins. Similarly, the lowest number of essential genes mapped to subcategory D, representing a positive correlation for slow growing bacteria. Furthermore, this shows that COG classifications have the potential to be used as essential gene prediction algorithms, alongside other prediction methods such as strand-bias.

#### Codon adaptation index of *M. ulcerans* essential genes

The preference for synonymous codons among prokaryotes is now accepted to be the result of mutational bias and natural selection acting at the level of translation. Several studies that have focused on essential gene identification by computational methods, have used CAI values as measures of gene essentiality, in combination with other features. The CAI values of genes in a genome range between 0 and 1. A higher CAI value usually suggests that the gene of interest is likely to be highly expressed and therefore essential. Therefore, a CAI >0.5 can be used as a threshold for selection of essential genes that are also highly expressed. We computed the CAI for 424 essential genes of *M. ulcerans*, using the ACUA program [42], which implements the Sharp and Li method of CAI calculation [43]. Non-synonymous codons and stop codons were excluded during calculation. The CAI values of *M. ulcerans* essential genes ranged from 0.516 to 0.829. Above the selection threshold of 0.5, all genes are considered highly expressing and likely to be essential (Table S3). However, similarly to strand-bias, CAI alone is a weak selection criterion for the identification of essential genes [44]. It can be best used in combination with other selection features, as we observed that *M. ulcerans* essential genes located on the leading strand have higher CAI values than those located on the lagging strand (Table S3). Similarly, CAI values can also be used in combination with COG classes, as we observed that essential genes associated with J, E, and H classes have higher CAI values than those associated with other classes (Table S3). We propose therefore that CAI, COG, and strand-bias can be used in combination, for the development of an effective algorithm for computational identification of essential genes.

#### Enzyme enrichment in *M. ulcerans* essential genes

It has recently been shown that enzymes are enriched in essential bacterial genes by overrepresented ligases and underrepresented oxidoreductases when compared to non-essential genes of same bacteria [45]. We did not perform a cross-comparison of enzyme types among essential and non-essential genes of *M. ulcerans*. Instead, we were interested to determine the specific enzyme class to which maximum number of essential enzymes belonged. To perform this analysis, the enzyme commission (EC) code annotations available as GBK files for *M. ulcerans* were retrieved from the NCBI FTP server (ftp://ftp.ncbi.nih.gov/genomes/Bacteria). In addition, information about EC numbers was also taken from NCBI, Swiss-Prot, and KEGG databases to cover the maximum possible number of enzymes. Among the 424 essential genes, EC numbers were available for 336 essential enzymes. Among these 336 essential enzymes, 128 were transferases, 65 oxidoreductases, 43 ligases, 43 hydrolases, 40 lyases, and 17 isomerases (Figure 3B and Table S3).

While performing this analysis, we became interested in determining whether enzymes from different classes also show strand-bias. Unlike for previous comparisons among two or more essential gene prediction features, such as COG categories and strand-bias [39], to the best of our knowledge, strand-bias among essential enzymes is not known. To determine any potential relationship between two these features, enzymes from each class were evaluated for their location on either strand. Interestingly, a significant level of bias towards the leading strand was observed in each class (Figure 3B and Table S3). We roughly propose that transferases and isomerases show maximum strand-bias. As in case of *M. ulcerans*, although only 17 isomerases were identified as essential, 15 of them were located on leading strand. As stated earlier, there were equal number of essential ligases (43/336) and hydrolases (43/336), the pattern of strand-bias exhibited by enzymes from these classes was also found to be similar i.e., there were 35 ligases and 35 hydrolases on leading strand and 8 ligases and 8 hydrolases on lagging strand. As strand-bias and enzyme classes distribution are now known to exist among essential genes, it would be worthwhile to further validate this relationship in those bacteria whose essential genes have already been identified experimentally (Table S1). Identification and further validation of this feature can provide an excellent opportunity to add additional parameter in essential genes identification algorithms.

It is known that some non-coding regions are also essential if they contain DNA sequences responsible for important biological functions. Such sequences include the chromosomal origins of replication, promoters, tRNAs, rRNAs, and small RNAs. It is likely that some essential genes were missed due to limitations in available experimental data about essential genes from other bacterial species. However, until large scale experimental gene essentiality studies are conducted for *M. ulcerans*, essential genes data from our study can serve as a reference. Furthermore, the post identification evaluation of essential genes, based on other genomics features, also supports the likelihood that the identified genes are essential for *M. ulcerans*.

### Identification of Non-host Essential Proteins

Unique pathways are those that are specific to the pathogen but absent in its host. Proteins in such pathways can also be considered unique to the pathogen and could serve as potential drug and vaccine targets. In addition, several unique or pathogen-specific proteins are known to be present in common pathways of the pathogen and host, as identified during our analysis for *M. ulcerans* (Table S2), and in several previous studies on other bacteria [13], [20], [22], [46]. Similarly, it has been observed that a single unique protein can be involved in multiple pathways. Proteins that are involved in more than one pathway could be more effective drug targets, when, in addition, they are non-host proteins. However, being unique, no homology to host proteins, and involvement in multiple metabolic pathways could not effectively serve as sole criteria for selection of favorable drug targets. It is quite possible that a bacterial protein might be involved in multiple metabolic pathways, show no similarity to human proteome, but that its disruption might not offer any therapeutic benefit. The reasons for this may include presence of paralogs, isoenzymes, and most importantly, being non-essential for the pathogen. Therefore, identification of bacterial proteins that regulate key factors, such as nutrient uptake, survival in the host environment, virulence, and pathogenicity, are of great importance for disruption of the pathogen’s functions and existence. Such proteins can be classified as essential for the pathogen [47]. However, it has also been observed that not all essential bacterial proteins are non-homologous to their hosts proteome [13], [22], [48]. Bacterial proteins that show no similarity to their hosts and also essential for the microbe can serve as highly effective therapeutic candidates. We therefore performed a cross-comparison between the 411 non-host proteins (Table S2) and 424 essential proteins (Table S3) of *M. ulcerans*, and shortlisted 236 proteins as both non-host and essential (Table S4). Metabolic pathway information was inferred for each, and 45 non-host essential proteins were mapped to unique pathways and 191 to common pathways (Table S4). These non-host essential proteins represent an attractive refined dataset that could be exploited for future drug design and vaccine production against *M. ulcerans.*

### Prioritization of Drug Targets

Previous studies using computational comparative/subtractive genomics have focused mainly on determining whether a non-human homolog is also an essential protein and in which pathway it is involved [20]–[22], [48]. Although we also considered these important criteria, advances in genome sequencing, bioinformatics, and cheminformatics, coupled with experimental data, have shown that there are several additional factors that can aid in determining the suitability of therapeutic targets. The most important of these are: preferred low MW (≤110 kDa); subcellular localization to determine the accessibility of a drug target; presence and absence of transmembranes; druggability; and availability of 3D structural information [9], [28], [49], [50]. Incorporation of such additional details can aid in improving the screening of therapeutic targets, as we have shown in a previous study of *M. genitalium* [13], and others have indicated in studies of computational identification of drug targets in different bacterial and fungal pathogens [9], [49]. Therefore, once the non-host essential protein dataset of *M. ulcerans* was defined (Table S4), we further characterized it following the above-mentioned drug target prioritization parameters.

It has been suggested that smaller proteins are more likely to be soluble and easier to purify [38]. The MW for each potential drug target was calculated using online tools and confirmed with the available literature. Among the 236 non-host essential proteins, 231 had MWs ≤110 kDa, indicating that these target proteins can be experimentally studied for drug development (Table S4).

Subcellular localization is a key functional attribute of a protein. Cellular functions are often localized in specific compartments; predicting the subcellular localization of unknown proteins could thus be used to obtain information about their functions, and to select proteins for further study. Moreover, studying the subcellular localization of proteins is also helpful in understanding disease mechanisms and developing novel drugs [51]. All bacterial proteins are synthesized in the cytoplasm, and most remain there to carry out their unique functions. Other proteins, however, contain export signals that direct them to other cellular locations. In Gram-positive bacteria, these include the cytoplasmic membrane, cell wall and extracellular space. In Gram-negative bacteria, they include the cytoplasmic membrane, the periplasm, the outer membrane and the extracellular space. In most cases the whole protein is located in a single compartment; however, proteins can also span multiple localization sites [52].

The subcellular localization of 236 non-host essential proteins of *M. ulcerans* was evaluated using the MycobacSVM server, and further crosschecked by TMHMM, and TOPCONS. Among these, 180 proteins were predicted to be cytoplasmic, 52 to be integral membrane proteins, and 4 were predicted as attached to the membrane (Table S4). Bacterial cell surface/membrane proteins and secreted proteins are of interest for their potential as vaccine candidates and diagnostic targets [53]. Therefore, identified membrane proteins of *M. ulcerans* have potential to act as common vaccine candidates, and may also be active against those bacterial species in which these proteins are also evolutionarily conserved or possess orthologs.

Druggability of each non-host essential protein of *M. ulcerans* was identified by sequence similarity to the targets of small-molecule drugs, using the DrugBank database. A local copy of the DrugBank database was downloaded and a BLASTP search was performed to align the non-host essential proteins with the list of drug-targeted proteins from DrugBank. This led to the identification of 89 *M. ulcerans* proteins that were highly similar to the binding partners of FDA- approved drugs, experimental small-molecule compounds, or nutraceutical compounds (Table S5); this supports the potential of comparative genomics in drug discovery. Furthermore, metabolic pathway analysis showed that among these 89 *M. ulcerans* proteins, 19 were from unique pathways and 70 were from common pathways (Table S5). This comparison with drug-targeted proteins produced a list of approved drug and drug-like compounds that bind to proteins with similar sequences to those of *M. ulcerans*. Although protein sequence similarity does not guarantee identical structures or binding pockets, it seems reasonable that careful filtering of this set could reveal a panel of potential binding compounds primed for optimization and derivatization using traditional medicinal chemistry [23]. This gives the interesting possibility of applying bioinformatics analysis to bypass a portion of the tedious *de novo* drug development pipeline.

Finally, we searched the non-host essential proteins of *M. ulcerans* for the presence of 3D structures and/or 3D structures complexed with a ligand, inhibitor or drug. Such structural information could greatly enhance the druggability value by facilitating a structure-based drug design, including homology modeling, docking, virtual screening or pharmacophore-based screening [49]. The 3D structural information for each of the non-host essential protein was retrieved from PDB and ModBase. Out of the 236 non-host essential proteins of *M. ulcerans*, none were identified as having experimentally determined 3D structure in PDB. However, 95 had 3D models in ModBase (Table S4). In order to avoid any ambiguity, we further cross-checked for the presence of available 3D models of *M. uclerans* proteins in PDB that we might have missed. This was done by performing a BLASTP search of *M. ulcerans* protein sequences against the PDB database, and manual keyword searches in PDB. This led to the identification of an experimentally determined 3D model of a *M. ulcerans* enzyme, cystathionine gamma-synthase, deposited under PDB ID: 3QI6. This enzyme is an essential enzyme of *M. ulcerans* and associated with cysteine and methionine metabolism, nitrogen metabolism, and sulfur metabolism pathways, as identified during our analysis (Table S4). However, based on more than 47% identity to human enzyme, cystathionine gamma-lyase (UniProt ID: P32929), it was not included in the *M. ulcerans* non-host essential proteins dataset. The presence of only one full length experimentally determined 3D structure of *M. ulcerans* protein in PDB is a matter of concern, and highlights the need for studies focused on structural characterization of *M. ulcerans* proteins to accelerate the drug discovery process at both computational and experimental levels.

### Drug Targets from Unique Metabolic Pathways of *M. ulcerans*

In the following sections, we discuss some of the most attractive therapeutic targets identified from the 29 pathogen specific metabolic pathways, along with their molecular mechanisms and involvement in critical metabolic steps. The selection of discussed drug targets were on the basis of following six criteria: (i) essential for pathogen, (ii) show no similarity to host proteins, (iii) their role in key metabolic pathways (pathways which are already known to be targeted for their therapeutic potential in different bacteria), (iv) subcellular localization analysis for accessibility, (v) druggability potential via DrugBank search, and (vi) availability of experimentally solved 3D structures or templates for computational drug discovery and modeling.

#### Bacterial secretion systems

Bacterial protein secretion pathways play a key role in modulating biotic associations as well as pathogenicity, via secretion of virulence factors. In Gram-negative bacteria, six general classes of secretion systems have been identified which show considerable diversity, and facilitate the entry of secreted proteins into host cells, modification of host physiology, and colonization. In Gram-positive bacteria, secreted proteins are commonly translocated across the single membrane by general secretion (Sec), and twin-arginine translocation (Tat) [54]. Although Gram-positive bacteria share some of the same secretion systems as Gram-negative bacteria, others such as *M. tuberculosis* and *M. marium*, which have a hydrophobic, nearly impermeable cell wall called the mycomembrane, have developed a specialized secretion system. This is known as the type VII secretion system and is responsible for the virulence and translocation of proteins across both the membrane and the cell wall [55], [56]. Although *M. ulcerans* also belongs to the genus *Mycobacteria* and is thought to have evolved from *M. marium*, it does not have a type VII secretion system; this is believed to be an immune evasion strategy of the pathogen [57]. The importance of bacterial secretion systems is well established in terms of both bacterial viability and pathogenicity and they have therefore been widely suggested as targets for new drugs, vaccines, and diagnostic markers. Several proteins from secretion pathways have been proposed as drug and vaccine targets using both computational and experimental approaches for different Gram-positive and Gram-negative bacteria, including *B. pseudomallei* [20], *S. aureus* [21], and *M. tuberculosis* [58]. No such information is yet available for *M. ulcerans*. We therefore evaluated the therapeutic potential of *M. ulcerans* secretion pathways via comparative genomics analysis. Initially, in accordance with the KEGG annotations, we identified 12 proteins from the *M. ulcerans* genome that mapped to protein export pathway (Table 1). Among these, eight were further identified as non-host (Table S2) and seven as essential (Table S3). Our focus was to select proteins that were non-host plus essential; six proteins were selected: preprotein translocase subunit (SecY), preprotein translocase subunit (SecE), inner membrane protein translocase component (YidC), preprotein translocase subunit (SecA), lipoprotein signal peptidase (LspA), and sec-independent protein translocase transmembrane protein (TatC) (Table S4).

SecE, SecY, SecA, and YidC are part of the *M. ulcerans* Sec pathway and TatC belongs to the Tat export pathway. In *Mycobacteria* including *M. ulcerans*, both these secretory pathways are functional and essential or indispensable. SecY, SecE, and SecG form an essential heterotrimeric protein complex that is central to the Sec pathway. This SecYEG complex serves as a transport channel for the movement of protein synthesized in the cytoplasm to the extracytoplasmic environment. This transport and movement of protein through the SecYEG channel is regulated by cytoplasmic ATPase SecA, via repeated cycles of ATP-binding and hydrolysis. SecG is expendable, although it increases the efficiency of protein export. It has been observed that disruption of SecYEG-SecA leads to aggregation of unfolded bacterial proteins in the cytoplasm and triggers a cellular stress response. LspA, also identified as a potential drug target (Table S4), is responsible for the cleavage of signal peptides from lipoproteins, thereby leading to the folding of proteins into mature conformations. These functional lipoproteins are known to be involved in the virulence of mycobacteria. Therefore, following the biological significance and increasing evidence supporting the therapeutic potential of protein export machinery, we suggest that the development of inhibitors against these proteins holds great therapeutic potential for the treatment of *M. ulcerans* infections.

#### Peptidoglycan biosynthesis

Cell walls are important and integral components helping bacteria to maintain their morphology as well as to withstand unfavorable conditions. The disruption of bacterial cell walls leads to cell lysis and hence cell death. Several antibiotics, such as penicillin, bacitracin, and vancomycin, kill bacteria by interfering with the biosynthesis of their cell walls [59]. Cell wall biosynthesis therefore remains a valid target for novel antibiotic development, especially for those drugs that can specifically inhibit any one of the series of essential enzymatic functions involved in the assembly of peptidoglycan (PG). To evaluate the therapeutic potential of the *M. ulcerans* PG biosynthesis pathway, we initially performed genome-wide metabolic pathway analysis, and identified 14 proteins from the *M. ulcerans* genome associated with PG biosynthesis. Of these, 12 (MurA, MurB, MurC, MurD, MurE, MurF, MurG, DacC, Ddl, MraY, UppP, and PbpB) were further identified as essential, mapped to COG subcategory M (cell wall biogenesis), and preferentially located on the leading strand, except for MurB. Following subcellular localization analysis, MurA–MurF and Ddl were identified as cytoplasmic, and MraY, UppP, PbpB, and DacC as membrane proteins. As this pathway is absent in humans, none of the 11 enzymes showed homology with human proteins, and they were therefore classified as non-host plus essential (Table S4). PG, which forms more than 70% of the weight of the cell wall, is a large molecule responsible for maintaining morphology and balance via osmotic pressure [60]. Biosynthesis of PG is a complex process of assembly and polymerization. Briefly, at the first assembly step, MurA and MurB catalyze the formation of UDP-GlcNAc-enolpyruvate and UDP-MurNAc, respectively, followed by the successive additions of L-alanine, D-glutamic acid, *meso*-diaminopimelic acid or L-lysine, and D-alanyl-D-alanine, to form UDP-MurNAc-L-Ala-γ-D-Glu-*meso*-A_2_pm-D-Ala-D-Ala. These steps are catalyzed by specific peptide ligases, designated MurC, MurD, MurE, and MurF. The second stage of PG biosynthesis involves transglycosylation and transpeptidation reactions of the disaccharide pentapeptide monomers, and takes place in the periplasmic space catalyzed by several membrane and periplasmic enzymes [61]. The therapeutic potential of targeting the above enzymes as drug targets is evident from several experimental studies conducted on different bacteria, including *M. tuberculosis*. We therefore propose that these enzymes can effectively be targeted against *M. ulcerans*. MurA, for instance, which catalyzes the condensation of phosphoenolpyruvate and UDP-N-acetylglucosamine, is a valid target of the antibiotic, fosfomycin [62]. However, one potential drawback of targeting MurA is the presence of two separate genes, *murA1* and *murA2*, that encode proteins with the same enzymatic activity in Gram-positive pathogens such as *S. aureus* and *Streptococcus pneumoniae*. In addition, differences in the active sites also make it difficult to develop MurA-specific antibiotics that could effectively inhibit both enzymes because the two homologs of the *murA* gene share less than 60% identity among Gram-positive species. Mutagenic studies have shown that disruption of either the *murA1* or the *murA2* gene had no significant effect on cell growth, but cells were unable to survive when both genes were removed [63]. To check whether *M. ulcerans* also contains two copies of *murA*, we performed a genome-wide search and found that *M. ulcerans* contains only one copy of *murA* gene (data not shown); meaning that the MurA of *M. ulcerans* is an attractive drug target for already available antibiotics. Another attractive drug target is D-Alanine:D-alanine ligase (Ddl); this catalyzes the ATP-driven ligation of two D-alanine molecules to form the D-alanyl:D-alanine dipeptide. This molecule is a key building block in PG biosynthesis and inhibition of this step leads to extensively weaker cell walls and cell death. D-cycloserine is a competitive inhibitor of Ddl, and is used as a second line of defense in the treatment of tuberculosis. We therefore suggest that fosfomycin and D-cycloserine should be tested as drugs of choice against the MurA and Ddl of *M. ulcerans*, respectively. Although not tested experimentally for *M. ulcerans*, based on genome-wide analysis, we suggest that antibiotics such as bacitracin and others of similar mechanism may not be good choices against *M. ulcerans* due to the presence of the *uppP* gene, known for encoding a protein responsible for conferring resistance to bacitracin.

#### Polyketide sugar unit biosynthesis

Polyketides are secondary metabolites that are formed as a result of the polymerization of acetyl and propionyl subunits. Polyketides play important roles in intercellular communication, maintenance of cell wall viability and defense mechanisms in different bacteria, including *M. ulcerans*, and potentially help through periods of desiccation [64]. Hence, the therapeutic potential of this pathway has been proposed in several studies [65], [66]. No such information is yet available for *M. ulcerans*. We identified four enzymes encoded by *rml* genes: Alpha-D-glucose-1-phosphate thymidylyl-transferase (RmlA), dTDP-glucose-4,6-dehydratase (RmlB), DTDP-4-dehydrorhamnose 3,5-epimerase (RmlC), and dTDP-6-deoxy-Llyxo-4-hexulose reductase (RmlD), associated with the polyketide sugar unit biosynthesis pathway. These four enzymes catalyze the steps of biosynthesis of dTDP-rhamnose from dTDP and glucose-1-phosphate. Following our comparative genomics analysis, each enzyme was also identified as essential, thereby highlighting the importance of targeting these enzymes as drug targets (Table S3). However, RmlA, RmlB, and RmlD showed more than 45% identity with human proteins. In contrast, RmlC showed no homology with human proteins. Furthermore, a search across DrugBank showed that *M. ulcerans* RmlC shares significant levels of identity with the binding partner (*M. tuberculosis* RmlC) of a small molecule experimental drug S,S-(2-hydroxyethyl) thiocysteine (Table S5). RmlC catalyzes a key step in dTDP-rhamnose synthesis by converting glucose-1-phosphate to dTDP-L-rhamnose via epimerizing the C3′ and C5′ positions of dTDP-6-deoxy-D-xylo-4 hexulose, making dTDP-6-deoxy-L lyxo-4-hexulose [67]. The essentiality of RmlC in mycobacterial survival is also evident from previous gene knock-out studies of *M. tuberculosis* and *M. smegmatis* [65]. We propose therefore that RmlC can serve as an effective drug target for disruption of *M. ulcerans* cell wall synthesis, for its essential and non-host nature, involvement in cell wall synthesis and, in comparison with other Rml enzymes, high substrate-specificity and cofactor-independent activity [65].

### Drug Targets from Common Metabolic Pathways of *M. ulcerans*

In addition to drug targets from pathogen-specific pathways, we identified several therapeutic targets from the common metabolic pathways of the host and pathogen (Table S4). Similarly to drug targets from unique pathways, these targets were found to be involved in multiple pathways and to be non-host plus essential. It is expected that targeting these proteins will lead to development of more potent antibiotics against *M. ulcerans*. Some of these attractive drug targets are discussed below.

#### Thiamine biosynthesis

We identified five drug targets from the thiamin biosynthesis pathway of *M. ulcerans*. These included the thiamine biosynthesis protein (ThiC), phosphomethylpyrimidine kinase (ThiD), thiamine biosynthesis oxidoreductase (ThiO), thiamine monophosphate kinase (ThiL) and thiazole synthase (ThiG) (Table S5). Thiamin (Vitamin B1) is an essential cofactor and is indispensable for the activity of the carbohydrate and branched-chain amino acid metabolic enzymes. The active form of thiamin is thiamin diphosphate (ThDP). Synthesis of bacterial ThDP is a two-step process involving the formation of the thiazole moiety, 4-methyl-5-β-hydroxyethyl thiazole phosphate (THZ-P) and the pyrimidine moiety, 4-amino-5-hydroxymethyl-2-methyl-pyrimidine pyrophosphate (HMP-PP) [68]. During the first step, THZ-P is derived from an oxidative condensation of tyrosine or glycine, cysteine and 1-deoxy-D-xylulose 5-phosphate via seven different enzymes including ThiG and ThiO. Parallel with this, formation of HMP-PP is accomplished in two steps regulated by ThiC and ThiD. In the second and final step, THZ-P and HMP-PP are coupled into the active form of thiamin (ThDP) through a final phosphorylation step mediated by ThiL [69]. *thiL* and other genes from the thiamin metabolism pathways of *Plasmodium falciparum* [70] and *M. tuberculosis* [71] have also been identified as essential for survival and as potential drug targets. Designing inhibitors of identified enzymes to block the biosynthesis of thiamin therefore represents an attractive strategy with potential to damage the growth and survival of *M. ulcerans*.

#### Aminoacyl-tRNA biosynthesis

Aminoacyl-tRNA synthetases (AaRS) are the group of enzymes that catalyze the acylation of amino acids to tRNA molecules in the translation stage of protein biosynthesis. Being identified as essential for the survival of the pathogen and playing a crucial role in protein biosynthesis, AaRS enzymes have received much attention for antibacterial drug discovery by several pharmaceutical companies and academic research groups [72]. In addition to essentiality, there are several features that favor targeting AaRS for drug discovery. These include: (i) the presence of considerable evolutionary divergence between prokaryotic and eukaryotic enzymes thus making them ideal candidates whose inhibition will not likely affect human enzymes [73], [74]; (ii) development of potent and broad spectrum antibiotics, as AaRs are highly conserved among pathogenic bacteria [73], [74]; (iii) the full complement of 20 synthetases is found in most bacterial pathogens and may represent 20 independent antibacterial targets [73], [74]; (iv) these enzymes are soluble, stable, and easy to purify in large quantities from recombinant expression systems, and can be assayed by one or more conventional methods amenable to high-throughput screening [75], [76]; and (v) the x-ray crystal structures for most of the synthetases are known from several bacteria, and provide a platform for rational drug design [73], [77].

We have highlighted three *M. ulcerans* AaRS as drug targets: arginyl-tRNA synthetase (ArgS), histidyl-tRNA synthetase (HisS), and phenylalanyl-tRNA synthetase (PheS) (Table S5). Although 18 AaRS were identified as essential for *M. ulcerans* (Table S3), 15 of these showed a considerable level of homology with human AaRS, and were therefore not included in further analysis. Currently, only one AaRS inhibitor, mupirocin, which inhibits bacterial IleS, is marketed as an antibacterial agent [78]. However, the IleS of *M. ulcerans* showed 48% identity with human IleS, and resistance to this agent is also widely reported; it may therefore not be an ideal choice. Recently, phenyl-thiazolylurea-sulfonamides have been identified as successful inhibitors of PheS in *Escherichia coli*, *Haemophilus influenzae*, *S. pneumoniae*, and *S. aureus*, showing high potency, broad-spectrum activity and selectivity for bacterial PheS versus the corresponding mammalian cytoplasmic and human mitochondrial enzymes [79]. It has also been observed that inhibition of these enzymes leads to disruption of protein biosynthesis, in turn resulting in the attenuation of bacterial growth under both *in vitro* and infectious conditions [80]. We propose therefore, that inhibition of ArgS, HisS, and PheS can lead to disruption of *M. ulcerans* protein synthesis with no side effects for its human host. It would be worthwhile to evaluate the antibacterial activity of phenyl-thiazolylurea-sulfonamides against the PheS of *M. ulcerans*.

#### Folate biosynthesis

Folate biosynthesis is an important biochemical pathway whose enzymes have been targeted since 1930 as key for antimicrobial therapy. Inhibition of two enzymes in this pathway, dihydropteroate synthase (FolP) and dihydrofolate reductase (DfrA), has been widely used for treatment of infections caused by bacteria such as *Pneumocystis carinii* [81], *T. gondii* [82], and protozoan parasite *P. falciparum* [83]. Recently, we have also reported that the DfrA of *M. genitalium* can serve as a potential therapeutic target [13]. The therapeutic potential of targeting the folate biosynthesis pathway of *M. ulcerans* is not well elucidated. The folate biosynthesis pathway of *M. ulcerans* comprises of 11 proteins. Among these, we identified four enzymes; DfrA, FolP, dihydroneopterin aldolase (FolB), and 6-pyruvoyl tetrahydrobiopterin synthase (PTPS) as non-host plus essential (Table S3). DfrA is a ubiquitous enzyme that is responsible for the reduction of dihydrofolate to tetrahydrofolate, an important co-factor in the biosynthesis of thymine. Inhibition of DfrA leads to cell death through lack of thymine as the cells have no alternative [84]. FolP catalyzes a condensation reaction yielding dihydropteroate, an intermediary metabolite subsequently converted to tetrahydrofolic acid, and essential for the syntheses of purine, thymidylate, glycine, methionine, pantothenic acid, and N-formylmethionyl-tRNA. In addition, it is an identified drug target in *P. falciparum* [83] and *T. gondii* [82]. As folate biosynthesis is a common pathway, significant homology between host and pathogen proteins can occur. However, none of the identified drug targets had human homolog. We suggest therefore that the DfrA and FolP of *M. ulcerans* hold strong therapeutic potential worthy of experimental follow-up.

#### Histidine biosynthesis

12 proteins from the *M. ulcerans* genome mapped to the histidine metabolism pathway, and out of these, eight were identified as non-host plus essential (Table S4). Druggability analysis also revealed several drug-like compounds that can be tested as inhibitors of the ATP phosphoribosyl transferase (HisG) and Histidinol dehydrogenase (HisD) of *M. ulcerans* (Table S5). HisG catalyzes the first committed step in histidine biosynthesis, condensation of ATP with phosphoribosyl pyrophosphate to produce phosphoribosyl ATP and inorganic pyrophosphate, leading to intermediates that play a role in purine biosynthesis. HisD regulates the final oxidation step of histidine synthesis. The therapeutic potential of HisG, HisD, and other enzymes from this pathway has been reported previously from comparative genomics in *M. tuberculosis* [85], *M. leprae* [19], *P. aeruginosa* [11], and *S. aureus* [21]. These predictions have recently been confirmed in a study in which nitrobenzothiazole-containing compounds were identified as successful inhibitors of *M. tuberculosis* HisG [86]. Similarly, in another recent study, several inhibitors of *S. aureus* histidine biosynthesis were computationally predicted and then confirmed experimentally [87]. These findings collectively highlights the therapeutic potential of the histidine metabolism pathway. We thus propose that *M. ulcerans* histidine metabolism pathway enzymes can serve as novel drug targets.

In addition to the drug targets discussed above, we identified several other novel targets from the common and unique pathways of *M. ulcerans* (Table S4). Homoserine dehydrogenase (ThrA) and homoserine kinase (ThrB), for example, are two enzymes involved in the glycine, homoserine, threonine, cysteine, and lysine metabolism pathways of *M. ulcerans* (Table S4). The therapeutic potential of both these enzymes has been confirmed in a recent study of *M. tuberculosis* [85]. In *M. ulcerans,* we identified both these enzymes as cytoplasmic, and non-host plus essential, suggesting that their inhibition holds therapeutic potential, as their successful disruption has been shown to deprive the pathogen of essential nutrients and synthesis of necessary components such as cell wall PG.

### Conclusions

We have performed comparative genomics analyses of the causative agent of BU, and have identified several proteins in the *M. ulcerans* genome that can be targeted for effective drug design and vaccine development. As many of the identified drug targets have been reported to play a role in the critical metabolic pathways that regulate bacterial pathogenicity and essential nutrient uptake, a systematic approach to develop drugs against these targets would likely be very promising for the treatment of BU. This information can lead to significant progress in testing the efficacy of already available antibiotics, in comparison with novel drug development, equally important but time consuming. It is expected that the drugs developed against identified targets will be specific to the pathogen and of minimal toxicity for the host. We are currently evaluating the therapeutic potential of these enzymes and have identified several inhibitors using virtual screening (data not shown, unpublished data), which we expect will greatly aid in development of novel inhibitors against *M. ulcerans*.

## Supporting Information

Table S1 **Experimentally determined essential genes of different bacteria from the Database of Essential Genes (DEG) version 6.8.**
(XLSX)

Table S2 **Non-host proteins of** ***M. ulcerans*** **identified from unique metabolic pathways and pathways common to** ***M. ulcerans*** **and humans.**
(XLSX)

Table S3 **Essential genes/proteins of** ***M. ulcerans*** **identified from unique metabolic pathways and pathways common to** ***M. ulcerans*** **and humans.**
(XLSX)

Table S4 **Non-host essential proteins of** ***M. ulcerans*** **identified as potential therapeutic candidates from unique metabolic pathways and pathways common to** ***M. ulcerans*** **and humans.**
(XLSX)

Table S5 **Non-host essential proteins of** ***M. ulcerans*** **similar to binding partners of FDA-approved drugs, experimental small molecule compounds, or nutraceutical compounds, as inferred from the DrugBank database.**
(XLSX)
