# Correction: Comparative Genomics Analysis of *Mycobacterium ulcerans* for the Identification of Putative Essential Genes and Therapeutic Candidates

**DOI:** 10.1371/annotation/8ce51727-91b0-463f-9b4c-768aab0a230a

**Published:** 2013-09-12

**Authors:** Azeem Mehmood Butt, Izza Nasrullah, Shifa Tahir, Yigang Tong

The affiliation given for the third author, Shifa Tahir, was incorrect. The correct affiliation is:

Department of Bioinformatics and Biotechnology, International Islamic University, Islamabad, Pakistan 

